# Expression and Localization Profiles of Rhoptry Proteins in *Plasmodium berghei* Sporozoites

**DOI:** 10.3389/fcimb.2019.00316

**Published:** 2019-09-10

**Authors:** Naohito Tokunaga, Mamoru Nozaki, Mayumi Tachibana, Minami Baba, Kazuhiro Matsuoka, Takafumi Tsuboi, Motomi Torii, Tomoko Ishino

**Affiliations:** ^1^Division of Molecular Parasitology, Proteo-Science Center, Ehime University, Toon, Japan; ^2^Division of Malaria Research, Proteo-Science Center, Ehime University, Matsuyama, Japan

**Keywords:** malaria, *Plasmodium*, merozoite, sporozoite, rhoptry, immuno-electron microscopy

## Abstract

In the *Plasmodium* lifecycle two infectious stages of parasites, merozoites, and sporozoites, efficiently infect mammalian host cells, erythrocytes, and hepatocytes, respectively. The apical structure of merozoites and sporozoites contains rhoptry and microneme secretory organelles, which are conserved with other infective forms of apicomplexan parasites. During merozoite invasion of erythrocytes, some rhoptry proteins are secreted to form a tight junction between the parasite and target cell, while others are discharged to maintain subsequent infection inside the parasitophorous vacuole. It has been questioned whether the invasion mechanisms mediated by rhoptry proteins are also involved in sporozoite invasion of two distinct target cells, mosquito salivary glands and mammalian hepatocytes. Recently we demonstrated that rhoptry neck protein 2 (RON2), which is crucial for tight junction formation in merozoites, is also important for sporozoite invasion of both target cells. With the aim of comprehensively describing the mechanisms of sporozoite invasion, the expression and localization profiles of rhoptry proteins were investigated in *Plasmodium berghei* sporozoites. Of 12 genes representing merozoite rhoptry molecules, nine are transcribed in oocyst-derived sporozoites at a similar or higher level compared to those in blood-stage schizonts. Immuno-electron microscopy demonstrates that eight proteins, namely RON2, RON4, RON5, ASP/RON1, RALP1, RON3, RAP1, and RAMA, localize to rhoptries in sporozoites. It is noteworthy that most rhoptry neck proteins in merozoites are localized throughout rhoptries in sporozoites. This study demonstrates that most rhoptry proteins, except components of the high-molecular mass rhoptry protein complex, are commonly expressed in merozoites and sporozoites in *Plasmodium* spp., which suggests that components of the invasion mechanisms are basically conserved between infective forms independently of their target cells. Combined with sporozoite-stage specific gene silencing strategies, the contribution of rhoptry proteins in invasion mechanisms can be described.

## Introduction

During the malaria lifecycle, three invasive forms, ookinetes, sporozoites, and merozoites, invade different types of cells in mosquito vectors and mammalian hosts. Among them, sporozoites, which transmit malaria disease from mosquitoes to mammalian hosts, firstly invade mosquito salivary glands prior to be injected into the mammalian skin during a blood meal. Sporozoites actively migrate through the skin to enter blood vessels and finally infect hepatocytes, where they develop into several thousand merozoites within a parasitophorous vacuole membrane (PVM). Merozoites invade erythrocytes by similarly forming a PVM, while ookinetes, the invasive form which develops in the midgut lumen after fertilization, simply traverse midgut epithelial cells without PVM formation.

The apical structures of invasive stage parasites in the phylum Apicomplexa are well-conserved as having microneme and rhoptry secretory organelles, which store proteins discharged prior to or post-invasion. Micronemes in the highly motile sporozoite and ookinete stages contain proteins involved in motility and cell traversal (Sultan et al., 1997; Yuda and Ishino, 2004; Kariu et al., 2006). In the case of the non-motile merozoite stage parasites micronemal proteins, such as merozoite surface proteins (MSPs), are involved in attachment to erythrocytes (Kadekoppala and Holder, 2010). Rhoptries are present only in infective stage parasites, such as *Plasmodium* merozoites and sporozoites which proliferate inside the PVM, and are absent in ookinetes, raising the possibility that rhoptry secretory proteins are involved in cell infection (reviewed in Baum et al., 2008; Frenal et al., 2017).

Rhoptry protein profiling has been conducted and characterized mainly in the related apicomplexans *Toxoplasma* tachyzoites and *Plasmodium* merozoites, and are classified into two protein groups based on their localization in rhoptry neck or rhoptry bulb (Bradley et al., 2005; Counihan et al., 2013). Rhoptry neck protein 2 (RON2), RON4, and RON5 are discharged as a complex prior to invasion and inserted into the target cellular membrane. The complex then interacts with apical merozoite protein 1 (AMA1) on the parasite plasma membrane to form a tight junction between the parasite and its target cell, a step which is essential for *Plasmodium* merozoite and *Toxoplasma* tachyzoite invasion of target cells (Alexander et al., 2005; Lebrun et al., 2005; Besteiro et al., 2009; Cao et al., 2009). Rhoptry bulb proteins are discharged subsequent to rhoptry neck proteins, to develop inside the parasitophorous vacuole. Many rhoptry blub proteins are species specific, in contrast to rhoptry neck proteins which are largely conserved between *Plasmodium* and *Toxoplasma*, such as components for the RON complex which is crucial for invasion by both parasites (Boothroyd and Dubremetz, 2008; Zuccala et al., 2012; Counihan et al., 2013; Kemp et al., 2013).

It has been studied whether tight junction formation mechanisms are critical for *Plasmodium* sporozoite invasion of mosquito salivary gland and mammalian hepatocyte target cells. This hypothesis is supported by the finding that the components of the RON complex, RON2, RON4, and RON5, are also expressed in sporozoites (Tufet-Bayona et al., 2009; Mutungi et al., 2014; Risco-Castillo et al., 2014). Moreover, a peptide inhibiting the interaction between AMA1 and RON2 reduced the *P. falciparum* sporozoite infection ability of cultured hepatocytes (Yang et al., 2017), and a conditional knockdown of RON2 or RON4 resulted in a reduction in sporozoite invasion ability (Giovannini et al., 2011; Ishino et al., 2019).

A comprehensive analysis of rhoptry proteins during sporozoite invasion using the sporozoite-stage specific knockdown system first requires the detailed profiling of rhoptry proteins in sporozoites. To date, about thirty rhoptry proteins have been classified in *P. falciparum* (reviewed in Counihan et al., 2013). In the present study, 12 proteins were selected as merozoite rhoptry proteins commonly expressed among *Plasmodium* spp.—i.e., expressed in both human and rodent malaria parasites—to examine their expression and localization in sporozoites. To achieve this goal, transgenic parasites were generated in *P. berghei* expressing target rhoptry proteins tagged with c-Myc tag at their C-terminus. Immuno-electron microscopy revealed that eight out of 12 candidate proteins are also localized to sporozoite rhoptries.

## Materials and Methods

### Parasites and Mosquitoes

A transgenic *Plasmodium berghei* ANKA parasite line was used in this study which constitutively expresses GFP under the control of the *elongation factor 1A* (*ef1*α) promoter without any drug resistance gene (Franke-Fayard et al., 2004), kindly given by Dr. Janse. Cryopreserved *P. berghei* ANKA infected erythrocytes were intraperitoneally injected into female ICR mice (4–6 weeks old, CLEA Japan, Tokyo, Japan) to obtain asexual stage parasites. To harvest mature schizonts, infected mouse erythrocytes with 0.5–1% parasitemia were cultured for 16 h and purified using Nycoprep 1.077 solution (Axis-Shield Diagnostics, Dundee, UK; Janse et al., 2006b). *Anopheles stephensi* SDA strain (*An. stephensi*) mosquitoes were maintained on a 5% sucrose solution during adult stages at 25°C. After feeding on *P. berghei* infected ICR mice, fully engorged mosquitoes were selected and kept at 20°C until dissection under a 12 h-light/12 h-dark cycle. All animal experimental protocols were approved by the Institutional Animal Care and Use Committee of Ehime University and the experiments were conducted according to the Ethical Guidelines for Animal Experiments of Ehime University.

### Real Time Reverse Transcription (RT)-PCR Analysis

Purified schizonts and infected-mosquito tissues (midguts and salivary glands) were collected in RNAlater (Thermo Fisher Scientific, San Jose, CA, USA) and stored at 4°C until RNA isolation. Total RNA was extracted using an RNeasy kit (Qiagen, GmbH, Hilden, Germany) and treated with DNaseI (Qiagen). Reverse transcription was conducted using a PrimeScript RT reagent Kit (Takara Bio, Otsu, Japan) with gDNA Eraser. Real-time RT-PCR reactions were performed using SYBR Premix Ex Taq (Takara Bio). The primer sequences used are listed in Supplementary Table S1. Real time PCR was performed using a TaKaRa PCR Thermal Cycler Dice (Takara Bio). Relative gene expressions were normalized by *ef1*α (PBANKA_1133300) mRNA levels and were compared using the delta, delta-Ct method (Pfaffl, 2001; Ishino et al., 2019).

### Generation of c-Myc-Tagged Rhoptry Protein Expressing Transgenic Parasites

To generate transgenic parasites expressing a rhoptry protein tagged with c-Myc at its C-terminus, the native locus of the targeted rhoptry molecule in the WT-GFP genome was replaced by single crossover homologous recombination with an expression cassette of the C-terminus of the rhoptry protein fused with a c-Myc tag, similar to the generation of RON2-c-Myc expressing parasites (Ishino et al., 2019). Schematic representation of the transgenic vector construction is shown in Supplementary Figure S1. Approximately 1,000–2,000 base pair of DNA fragments including the C-terminus of each rhoptry protein were amplified with specific primers (sequences of primers used in this study were listed in Supplementary Table S1) by PCR from genomic DNA of WT-GFP. Amplified PCR fragments of RAP1, RhopH1A, RhopH2, and RhopH3 were inserted into the pL0033 plasmid (BEI Resources, Manassas, VA, USA) at SacII and NcoI sites just before the c-Myc tag coding region, and these plasmids were then linearized at endogenous HpaI, SpeI, and XbaI sites, respectively (see Supplementary Figure S1A). RON5 and RALP1 fragments were inserted into an NdeI site disrupted pL0033 vector, which was linearized at an endogenous NdeI site (see Supplementary Figure S1B). To introduce XbaI recognition sites for linearization into the PCR fragments of RON3 and RON4, site directed mutagenesis was performed to introduce mutations without amino acid substitution as follows: RON3, 5716A > T and 5717G > C; and RON4, 1711T > C. Using the same strategy, the endogenous NcoI site in the amplified RON6 fragment was disrupted, to avoid interference with ligation into the SacII and NcoI sites of pL0033 (1878C > A). These DNA fragments were inserted into the pL0033 plasmid at SacII and NcoI sites, which were linearized at an introduced XbaI site for RON3 and RON4, and at an endogenous BamHI site for RON6 (see Supplementary Figure S1C). Electroporation of 10–15 μg linearized DNA into schizont-enriched WT-GFP and selection of transgenic parasites were performed as described (Janse et al., 2006a). DNA integration occurs at the target locus in the WT-GFP genome by single crossover homologous recombination as illustrated in Supplementary Figure S2. DNA integration into the target locus was confirmed by PCR genotyping and transgenic parasites were cloned by limiting dilution.

### Antibody Production

DNA fragments encoding amino acids 25–694 of ASP/RON1 and 899–1,072 of RON3 were amplified from *P. berghei* schizont cDNA by PCR and inserted into pEU-E01-GST-(TEV)-N1 (CellFree Sciences, Matsuyama, Japan) at EcoRV and BamHI sites to produce recombinant ASP/RON1 proteins and at XhoI and BamHI sites to produce recombinant RON3 with GST tag at their N-terminus. The GST-tagged recombinant proteins were produced using the wheat germ cell-free protein expression system (CellFree Sciences) and purified using a glutathione-Sepharose 4B column (GE Healthcare UK, Buckinghamshire, UK; Tsuboi et al., 2008). Purified GST-tagged recombinant proteins were used for immunization of Japanese white rabbits with Freund's adjuvant. Immunizations were done three times at 3-week intervals with 250 μg recombinant protein and the antisera were collected 14 days after the last immunization (Kitayama Labes, Ina, Japan). Anti-RON2 antibodies and anti-RAMA antibodies used in the study were prepared previously (Ishino et al., 2019).

### Western Blotting Analysis

Purified schizonts were treated with 0.08% saponin for 15 min on ice and the schizont pellets were resuspended in sample buffer solution for SDS-PAGE (nacalai tesque, Kyoto, Japan) containing 5% 2-mercaptoethanol. Sporozoites collected from midguts of infected mosquitoes at days 24–26 post-feeding were purified by density gradient centrifugation using 17% Accudenz solution (Accurate Chemical & Scientific Corporation, NY, United States; Kennedy et al., 2012). Sporozoite pellets were resuspended in sample buffer containing 5% 2-mercaptoethanol. Proteins were separated by SDS-PAGE using 5–20% gradient acrylamide gels (ATTO, Tokyo, Japan) and electroblotted onto polyvinylidene difluoride (PVDF) membranes. The PVDF membranes were blocked with Blocking One (nacalai tesque) overnight at 4°C and then incubated with primary antibodies (1:100 anti-c-Myc rabbit antibodies (A-14), Santa Cruz Biotechnology, Santa Cruz, CA, USA; 1:2,500, anti-ASP/RON1, RON3, or RAMA rabbit antibodies) diluted in PBS containing 0.01% Tween-20 (PBST) for 2 h at room temperature. After washing with PBST, the membranes were incubated with secondary antibodies conjugated to horseradish peroxidase (HRP; 1:30,000, Biosource, Camarillo, CA, USA) for 30 min at room temperature. Chemiluminescence detection was performed by adding Immobilon Western Chemiluminescent HRP Substrate (Merck Millipore, Darmstadt, Germany), and the signal was detected using ImageQuant LAS 4000 (GE Healthcare UK).

### Indirect Immunofluorescence Assay

Thin smears of purified schizonts were prepared on glass slides and fixed with cold acetone for 3 min. Sporozoites collected from midgut and salivary gland at day 24–26 post-feeding were seeded on 8-well multi-well slides, then air-dried and fixed with cold acetone for 3 min. The slides of schizonts and sporozoites were blocked with PBS containing 10% fetal calf serum at 37°C for 30 min and incubated with primary antibodies (1:50 for anti-c-Myc mouse monoclonal antibodies (9E10), 1:100 for anti-c-Myc rabbit antibodies (A-14), and 1:200 for anti RON2, ASP/RON1, and RAMA rabbit polyclonal antibodies) in blocking solution at 37°C for 2 h, followed by Alexa Fluor 488–conjugated goat anti-rabbit IgG and Alexa Fluor 568-conjugated goat anti-mouse IgG (Thermo Fisher Scientific) at 37°C for 30 min. Nuclei were stained with 1 mg/ml of 4′,6-diamidino-2-phenylindole (DAPI). The samples were mounted in ProLong Gold antifade reagent (Thermo Fisher Scientific) and observed with an inverted fluorescence microscope (Axio Observer Z1, Carl Zeiss, Oberkochen, Germany).

### Immuno-Transmission Electron Microscopy

Cultivated schizonts of WT-GFP or transgenic parasites expressing c-Myc tagged rhoptry proteins were purified by density gradient centrifugation. Infected midguts (day 17 or 21 post-feeding) or salivary glands (day 24 or 26 post-feeding) were dissected. The samples were fixed in 1% paraformaldehyde, 0.2% glutaraldehyde and embedded in LR-White resin (Polyscience, PA, USA). Ultrathin sections were blocked in PBS containing 5% non-fat dry milk and 0.01% Tween 20 (PBS-MT), then incubated at 4°C overnight with anti-c-Myc antibody (Santa Cruz biotechnology) or specific antibodies against ASP/RON1 (1:25), RAMA (1:100), or RON3 (1:200). The sections were washed with PBS containing 0.4% Block Ace (Yukijirushi, Tokyo, Japan) and 0.01% Tween 20 (PBS-BT), and the grids were incubated for 1 h at 37°C with goat anti-rabbit IgG conjugated with 15 nm gold particles (GE Healthcare) diluted 1:20 in PBS-MT, rinsed with PBS-BT, and fixed in 2% glutaraldehyde for 10 min at 4°C to stabilize the gold particles. The sections were then stained with 2% uranyl acetate in 50% methanol and lead citrate. Samples were examined using a transmission electron microscope (JEM-1230; JEOL, Tokyo, Japan).

### Gene IDs

The sequence information of genes in this article can be found in the PlasmoDB database (PlasmoDB.org) under the following gene ID numbers: *ron2*, PBANKA_1315700; *ron4*, PBANKA_0932000; *ron5*, PBANKA_0713100; *ron6*, PBANKA_0311700; *ralp1*, PBANKA_0619700; *asp/ron1*, PBANKA_1003600; *rap1*, PBANKA_1032100; *ron3*, PBANKA_1464900; *rama*, PBANKA_0804500; *rhoph1a*, PBANKA_1400600; *rhoph2*, PBANKA_0830200; and *rhoph3*, PBANKA_0416000.

## Results

### Selection of *Plasmodium* Rhoptry Proteins

In this study a rodent malaria parasite line, *P. berghei* ANKA strain expressing GFP under the control of *ef1*α promoter (WT-GFP; Janse et al., 2006a), was used to characterize rhoptry proteins in both merozoites and sporozoites. From the catalog of known *P. falciparum* rhoptry proteins (Counihan et al., 2013), 12 molecules whose orthologous genes exist in *P. berghei* were selected to compare their expression between merozoites and sporozoites. RON2, a sporozoite rhoptry protein demonstrated as transcribed predominantly in oocyst-derived sporozoites (Ishino et al., 2019), was included as a positive control. Among 12 selected proteins, six are conserved across the Apicomplexa phylum and another six are specific to *Plasmodium* spp. Gene IDs in *P. berghei* together with those of the orthologous genes in *P. falciparum* and in *Toxoplasma gondii* are listed in Table 1 (Aurrecoechea et al., 2009).

**Table 1:** The list of rhoptry proteins examined in this study.

	***P. berghei***		***P. falciparum***		***T. gondii***	**References**
	**ID**	Expression in sporozoite^*^	**ID**	**Merozoite**	**ID**	
RON2	PBANKA_1315700	Rhoptry	PF3D7_1452000	Neck	TGME49_300100	Cao et al., 2009; Ishino et al., 2019
RON4	PBANKA_0932000	Rhoptry	PF3D7_1116000	Neck	TGME49_229010	Richard et al., 2010
RON5	PBANKA_0713100	Rhoptry	PF3D7_0817700	Neck	TGME49_311470	Richard et al., 2010
RON6	PBANKA_0311700	Apical end	PF3D7_0214900	Neck	TGME49_297960	Proellocks et al., 2009
RALP1	PBANKA_0619700	Rhoptry	PF3D7_0722200	Neck	No ortholog	Haase et al., 2008;Ito et al., 2013
ASP/RON1	PBANKA_1003600	Rhoptry	PF3D7_0405900	Neck	TGME49_310010	Srivastava et al., 2010
RON3	PBANKA_1464900	Rhoptry	PF3D7_1252100	Bulb	TGME49_223920	Ito et al., 2011
RAP1	PBANKA_1032100	Rhoptry	PF3D7_1410400	Bulb	No ortholog	Riglar et al., 2011
RAMA	PBANKA_0804500	Rhoptry	PF3D7_0707300	Bulb	No ortholog	Topolska et al., 2004
RhopH1A	PBANKA_1400600	Not detected	3 paralogues	Bulb	No ortholog	Kaneko et al., 2001
RhopH2	PBANKA_0830200	Not detected	PF3D7_0929400	Bulb	No ortholog	Counihan et al., 2017
RhopH3	PBANKA_0416000	Not detected	PF3D7_0905400	Bulb	No ortholog	Sherling et al., 2017

* *Localization in sporozoites determined by IEM (except for RON6 which was detected by IFA) in this study*.

### Expression Profiling of Rhoptry Molecules in Schizonts and Developing Sporozoites

Firstly, the mRNA expression levels of the selected genes during sporozoite development in mosquito bodies were compared by real-time RT-PCR analysis with transcript levels in schizonts. Sporozoites, formed within oocysts on midguts of mosquitoes, are released into hemolymph and then invade salivary glands prior to being inoculated into mammalian skin with saliva. Since *P. berghei* sporozoite formation inside oocysts starts at days 10–14 post-feeding (Thathy et al., 2002; Ferguson et al., 2014), parasite-infected midguts were collected at days 9, 13, and 17 post-feeding. In addition, salivary glands of infected mosquitoes were collected at day 17 post-feeding. As a control, schizont-rich infected erythrocytes were purified and harvested. Relative mRNA amounts of selected genes were examined by real-time RT-PCR, normalized by *ef1α* mRNA expression.

**Figure 1:**
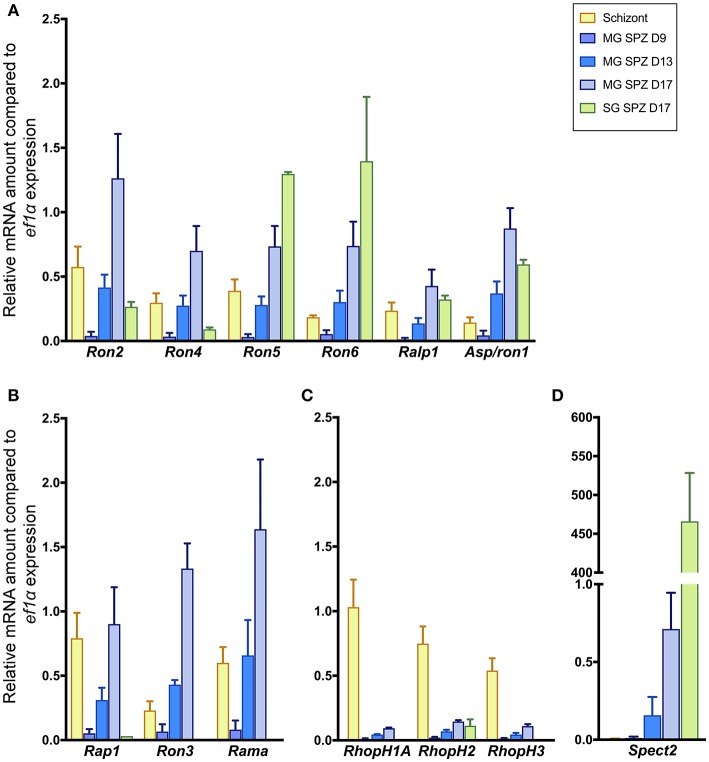
Transcriptional analyses of rhoptry genes in sporozoites and schizonts. Total RNA was extracted from parasite-infected mosquito midguts at days 9, 13, and 17 post-feeding (MG SPZ D9, MG SPZ D13, and MG SPZ D17); and salivary glands at day 17 post-feeding (SG SPZ D17). Total RNA was also extracted from schizont-enriched infected erythrocytes (Schizont). The mean values of relative mRNA amounts of each molecule, normalized by *ef1α* mRNA expression, are plotted as bar graphs with standard deviations from three independent experiments. **(A)** A group of molecules categorized as rhoptry neck proteins in merozoites. All examined molecules are transcribed in both merozoites and sporozoites. **(B)** A group of molecules categorized as rhoptry bulb proteins in merozoites whose transcriptions are detected in both merozoites and sporozoites. **(C)** A group of molecules categorized as rhoptry bulb proteins in merozoites whose transcriptions occur dominantly in merozoites. **(D)** Transcription profile of a typical micronemal protein, SPECT2, required for sporozoite migration toward hepatocytes after inoculation in the skin. Its transcript level drastically increases after sporozoite invasion of salivary glands.

Six molecules categorized as encoding rhoptry neck proteins in merozoites (*ron2, ron4, ron5, ron6, rhoptry-associated leucine zipper-like protein 1 (ralp1)*, and *apical sushi protein* (*asp*)/*ron1*) are also transcribed in sporozoites (Figure 1A). Among six genes encoding rhoptry proteins localized to the bulb region in merozoites, *rhoptry-associated protein 1* (*rap1), ron3*, and *rhoptry associated membrane antigen* (*rama)* are also transcribed in sporozoites (Figure 1B); while the other three, encoding the components of the high-molecular mass rhoptry protein complex (RhopH complex; Kaneko et al., 2001, 2005; Ling et al., 2003, 2004; Vincensini et al., 2008; Comeaux et al., 2011; Nguitragool et al., 2011; Counihan et al., 2017; Ito et al., 2017; Sherling et al., 2017), are transcribed far less in sporozoites than in schizonts (Figure 1C). This data raises the possibility that RhopH1A, RhopH2, and RhopH3 may play roles predominantly in merozoites. In contrast, *ron5, ron6, asp*/*ron1*, and *ron3* are predominantly transcribed in sporozoites vs. schizonts. The transcripts of rhoptry molecules expressed in sporozoites increase during sporozoite maturation in oocysts. After sporozoite invasion of salivary glands, the transcript amounts of *ron2, ron4, rap1, ron3*, and *rama* are significantly decreased, while transcripts of *ron5, ron6, ralp1*, and *asp1/ron1* remain high or increase. In salivary gland sporozoites, *ron5* and *ron6* are the highest transcribed among rhoptry molecules; however, their amounts remain ~200-fold less than that of a micronemal molecule, *sporozoite protein essential for cell traversal 2* (*spect2;* Ishino et al., 2005), whose transcription is strongly enhanced after sporozoites invade salivary glands (Figure 1D). These results demonstrate that most merozoite rhoptry molecules, except for those encoding RhopH complex components, are expressed in both infective stages, merozoites and sporozoites.

### Expression of Rhoptry Proteins in Sporozoites

To comprehensively examine protein expression patterns of rhoptry proteins in merozoites and sporozoites, transgenic parasite lines were generated by single-crossover homologous recombination to express each rhoptry protein fused with a C-terminal c-Myc tag (see Material and methods). Ten transgenic parasite lines were successfully isolated. Specific antibodies against recombinant protein were prepared for ASP/RON1 and RAMA, since they are predicted to have C-terminal glycosylphosphatidylinositol (GPI) anchored domains (Gilson et al., 2006) and therefore the modification of their C-terminal structure might disrupt their function (see Supplementary Figure S3). Specific antibodies against the middle region of RON3 were also prepared, because it was demonstrated that a 40 kDa fragment of C-terminal RON3 is cleaved during schizont maturation in *P. falciparum* (Ito et al., 2011).

**Figure 2:**
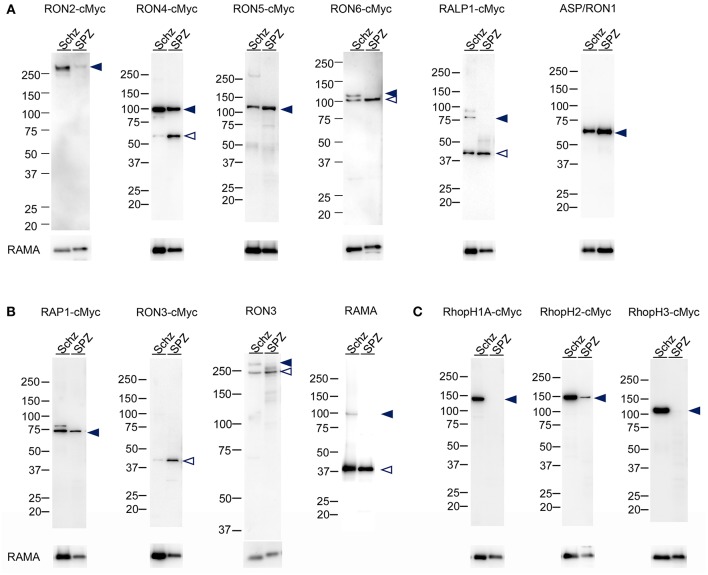
Western blot analyses of rhoptry proteins in schizonts and sporozoites. Proteins were extracted from schizonts purified after *in vitro* culture of parasite infected erythrocytes (Schz) or sporozoites purified from midguts at days 24–26 post-feeding (SPZ). Each lane contains proteins from 1.5 × 10^5^ schizonts or sporozoites of transgenic parasites expressing c-Myc tagged rhoptry proteins or WT-GFP. Target proteins were detected by western blotting using anti c-Myc antibodies or specific antibodies against ASP/RON1, RON3, or RAMA. The transgenic parasite lines used as antigens are indicated above panels; for example, RON2-cMyc. When specific antibodies were used to detect target molecules in WT-GFP parasites, the name of the target molecule is instead indicated. Closed- and open- arrowheads demonstrate the expected full-length and cleaved target proteins, respectively. The sizes of protein markers (kDa) are indicated on the left of each image. **(A)** Expression patterns of proteins categorized as rhoptry neck proteins in merozoites. ASP/RON1 was detected using rabbit specific antibodies as it contains a C-terminal GPI-anchor domain and is likely refractory to C-terminal c-Myc integration. **(B)** Expression patterns of proteins which are categorized as rhoptry bulb proteins in merozoites and transcribed in both merozoites and sporozoites. In the case of RON3, anti-c-Myc antibodies only recognized a roughly 40 kDa protein (RON3-cMyc), demonstrating that the C-terminal region was cleaved in both merozoites and sporozoites. Specific anti-RON3 antibodies detected full-length and cleaved forms of RON3 (RON3). **(C)** Expression patterns of proteins which are categorized as rhoptry bulb proteins in merozoites and predominantly transcribed in schizonts. Consistent with transcription analyses, the protein amounts of RhopH1A, RhopH2, and RhopH3 are far less in sporozoites than those in merozoites, indicated by closed arrowheads.

Protein lysates of 1.5 × 10^5^ schizonts and oocyst-derived sporozoites of each transgenic parasite line expressing c-Myc tagged rhoptry protein or WT-GFP were analyzed by western blotting using anti-c-Myc antibodies or specific antibodies against ASP/RON1, RAMA, and RON3. In schizonts all examined c-Myc tagged rhoptry proteins, except for RON3, were detected at the expected size of full-length (indicated by closed arrowheads, Figures 2A,B), demonstrating that c-Myc fused rhoptry proteins are successfully expressed. In addition, the processed forms of RON4 and RALP1 were detected at ~60 and 40 kDa (indicated by open arrowheads). In the case of RON3, anti-c-Myc antibodies detected ~40 kDa fragment as reported in *P. falciparum*, while anti-RON3 antibodies recognized two bands, near 250 kDa, corresponding to the full-length and processed RON3. It was confirmed that a roughly 40 kDa fragment of the C-terminal region in RON3 is cleaved in *P. berghei* mature schizonts as well as in *P. falciparum*. Antibodies against ASP/RON1 and RAMA recognized corresponding proteins at the size of expected full- and processed-proteins, confirming the specificity of these antibodies.

RhopH1A and RhopH3 proteins were not detected in sporozoites, while RhopH2 was detected as a far weaker band compared to that in schizonts, which is in good agreement with the transcriptional data (Figure 2C). In addition, RON2 production in sporozoites was significantly less than in schizonts. Proteolysis patterns are conserved between schizonts and sporozoites, although the ratio of uncleaved protein is less in oocyst-derived sporozoites than in schizonts. Since it takes longer for sporozoite maturation in oocysts than merozoites in schizonts, proteolysis of rhoptry proteins might be enhanced in sporozoites.

### Protein Localization of Rhoptry Molecules in Merozoites and Sporozoites

**Figure 3:**
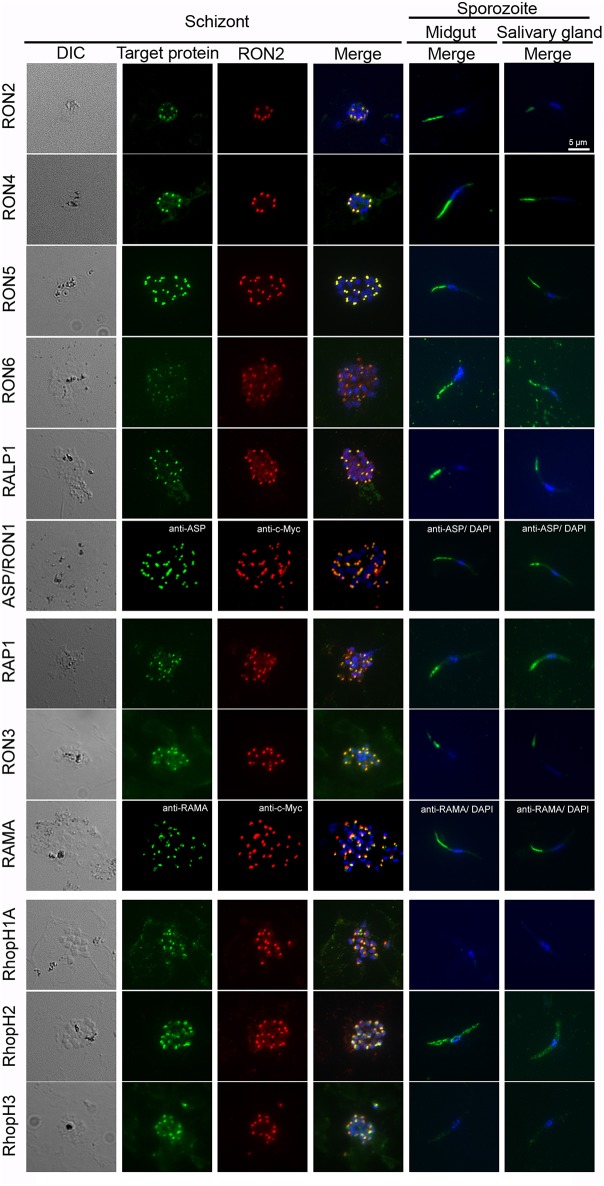
Expression pattern of rhoptry proteins in merozoites and sporozoites. Schizonts and sporozoites collected from midguts or salivary glands at day 24–26 post-feeding were fixed with acetone on glass slides. In schizonts, anti-c-Myc antibodies were used to detect target c-Myc fused rhoptry protein (shown in green) in each transgenic parasite, which is compared with the localization pattern of RON2 (shown in red) and nuclei (blue) stained by anti-RON2 antibodies and DAPI, respectively. To detect ASP/RON1 and RAMA, RON2-c-Myc transgenic parasites were used as antigens and target proteins and RON2 was detected by specific antibodies (green) and anti-c-Myc antibodies (red). Differential interference contrast (DIC) images are shown in the left panels. In sporozoites (right two panels), the localization of target proteins (green) and nuclei (blue) are shown in the merged images. Bar indicates 5 μm.

Rhoptry proteins in *P. falciparum* merozoites and *T. gondii* tachyzoites are categorized according to their detailed localization in rhoptries; specifically, rhoptry neck proteins (RON2, RON4, RON5, RON6, ASP/RON1, and RALP1) and rhoptry bulb proteins (RON3, RAP1, RAMA, RhopH1A, RhopH2, and RhopH3) (Counihan et al., 2013; Kemp et al., 2013). All rhoptry proteins examined in this study were confirmed to localize to the apical end region of *P. berghei* merozoites, similar to the RON2 marker signal, by immunofluorescent assay (IFA) using anti-c-Myc or specific antibodies (Figure 3, left columns). By comparison to RON2 localization, RON4, RON5, RON6, ASP/RON1, and RALP1 were suggested to be localized to the rhoptry neck region. This indicates that the C-terminal c-Myc tagging does not interfere with proper rhoptry protein localization. Additionally, their localization was examined in sporozoites collected from midguts and salivary glands (Figure 3, right columns). Specific signals corresponding to RhopH1A and RhopH3 were barely detected in sporozoites by IFA, confirming the western blotting data. The signal for RhopH2 was detected as a diffuse pattern in the cytoplasm of sporozoites. Taking into consideration the western blotting result, a small amount of RhopH2 is also expressed in sporozoites; however, it is not localized to rhoptries. Other examined proteins were detected at the apical end of sporozoites from both midguts and salivary glands, demonstrating that these proteins were transported to the apical region during sporozoite formation in oocysts and maintained even after sporozoite invasion of salivary glands.

### Eight Proteins Are Localized to Rhoptries in Sporozoites

**Figure 4:**
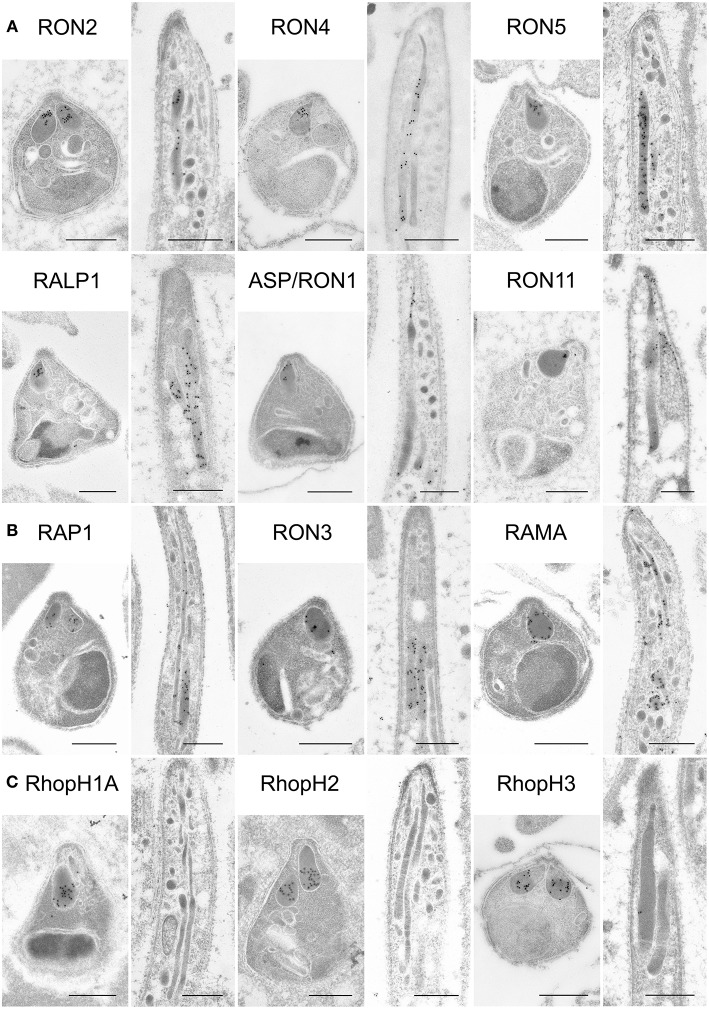
Localization analyses of rhoptry proteins in merozoites and sporozoites by immuno-electron microscopy. Longitudinally sectioned merozoites in cultivated schizonts and oocyst-derived sporozoites (at day 17 or 21 post-feeding) of each transgenic parasite line or WT-GFP were prepared for IEM analyses. Target proteins were detected by incubation with anti-c-Myc rabbit antibodies or specific antibodies against ASP/RON1 or RAMA, followed by secondary antibodies conjugated to gold particles. **(A)** A group of rhoptry proteins categorized as rhoptry neck proteins in merozoites. All examined proteins are confirmed to be localized to the rhoptry neck region in *P. berghei* merozoites. In contrast, most of them, except ASP/RON1, are distributed throughout rhoptries in sporozoites, as demonstrated previously regarding RON11. **(B)** A group of proteins expressed both in merozoites and sporozoites and categorized as rhoptry bulb proteins in merozoites. It is noteworthy that RAMA localizes to the rhoptry membrane in merozoites. In sporozoites, they are distributed in the rhoptry body region. **(C)** A group of proteins categorized as rhoptry bulb proteins in merozoites, which are produced predominantly in merozoites. IEM confirmed that none of them, RhopH1A, RhopH2, and RhopH3, are localized to rhoptries in sporozoites, despite their clear localization to the rhoptry bulb in merozoites. Bars, 500 nm.

To determine the precise localization of rhoptry proteins, immuno-electron microscopy (IEM) was performed using schizont stage merozoites and oocyst sporozoites. In *P. berghei* merozoites, RON2, RON4, RON5, RALP1, and ASP/RON1, which are categorized as rhoptry neck proteins in *Pf* merozoites, were confirmed to localize to the rhoptry neck region (Figure 4A). In addition, RAP1, RON3, RhopH1A, RhopH2, and RhopH3 are observed in the rhoptry bulb region, as reported for *Pf* merozoites (Figure 4B). RAMA is observed on the rhoptry membrane at the bulb region. RON6 could not be detected by anti-c-Myc antibodies, possibly because its protein amount in merozoites is not sufficient to be observed by IEM. This is the first comprehensive demonstration of rhoptry protein localization in *Plasmodium* merozoites.

**Figure 5:**
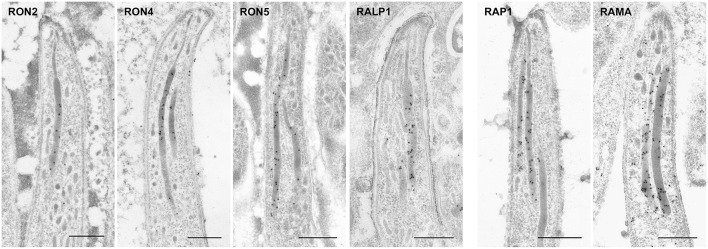
Localization of rhoptry proteins in sporozoites residing in mosquito salivary glands. Salivary glands of parasite infected mosquitoes were harvested at day 24 or 26 post-feeding and fixed for IEM. Target proteins were detected by anti-c-Myc antibodies or specific antibodies against ASP/RON1 or RAMA. All examined rhoptry proteins, despite their sub-localization in merozite rhoptries, are distributed throughout rhoptries in sporozoites residing in salivary glands, as well as oocyst-derived sporozoites. Bars, 500 nm.

In sporozoites formed inside oocysts, it was confirmed that three components for the RhopH complex do not accumulate in rhoptries, as expected from the observation of far less amounts of transcripts and proteins in sporozoites compared to merozoites (Figure 4C). Other than the RhopH complex, all proteins examined are localized to rhoptries in sporozoites as well as in merozoites. However, most proteins are distributed throughout rhoptries in sporozoites, despite their sub-localization in merozoites, suggesting that sub-compartmentation in rhoptries might be different between merozoites and sporozoites. This is consistent with the observation that the sub-localization of RON11 in rhoptries differs between merozoites and sporozoites (Figure 4A, Bantuchai et al., 2019). Only ASP/RON1 tends to accumulate in the thinner part in rhoptries near the tip of sporozoites. It is not clear whether RAMA localizes to the rhoptry membrane in sporozoites as in merozoites, as the maximum width of rhoptries is shorter in sporozoites than in merozoites. Matured sporozoites are released into the haemocoel followed by invasion of salivary glands. It was reported that, although morphologically similar, sporozoites in salivary glands show higher infectivity to the liver than sporozoites developed inside oocysts (Vanderberg, 1975); and accordingly transcription of some genes are upregulated or downregulated after sporozoite invasion of salivary glands (Kaiser et al., 2004; Mikolajczak et al., 2008; Tarun et al., 2008; Zhang et al., 2010). To determine rhoptry protein localization in liver infective sporozoites, salivary glands of transgenic parasites or WT-GFP infected mosquitoes were fixed for IEM analyses (Figure 5). RON2, RON4, RON5, RALP1, RAP1, and RAMA were detected in rhoptries of sporozoites residing in salivary glands, indicating that rhoptry proteins reside in rhoptries after sporozoite invasion of salivary glands, presumably available for subsequent invasion of hepatocytes in mammalian hosts. ASP/RON1 and RON3 could not be detected by anti-ASP/RON1 or anti-c-Myc antibodies, which might due to less target or c-Myc tagged protein amounts in salivary gland sporozoites.

## Discussion

In this study it is demonstrated that most rhoptry proteins reported in merozoites are expressed in sporozoites, with the exception of components of the RhopH complex. Together with three reported proteins, RAP2/3, RON11, and RON12 (Tufet-Bayona et al., 2009; Bantuchai et al., 2019; Oda-Yokouchi et al., 2019), nine rhoptry proteins are demonstrated to be commonly expressed in the infective stages of *P. berghei*. Our results on mRNA expression in schizonts and sporozoites of rhoptry molecules are mostly confirmed by single cell RNA-seq analyses in *P. berghei* (Reid et al., 2018). The orthologs of these proteins in *P. yoelii*, another rodent malaria parasite, are transcribed in oocyst-derived sporozoites (Tarun et al., 2008) and their proteins, except for RALP1, were detected in salivary gland residing sporozoites (Lindner et al., 2013). In addition, the orthologous proteins in the human malaria parasite *Plasmodium falciparum* were detected in salivary gland residing sporozoites (Lindner et al., 2013). Taken together, our finding that the most rhoptry proteins, except for RhopH complex components, are expressed in both merozoites and sporozoites indicates conservation of expression among *Plasmodium* species.

It is noteworthy that all examined commonly expressed rhoptry proteins localize to the neck region in merozoites, that is RON2, RON4, RON5, RALP1, ASP1/RON1, RON11, and RON12; while some rhoptry bulb proteins, RhopH1A, RhopH2, and RhopH3, are expressed predominantly in merozoites. The orthologs of proteins localized to the rhoptry neck are highly conserved across the infective stages of apicomplexan parasites, such as *Plasmodium, Toxoplasma, Babesia*, and *Cryptosporidium*; however, proteins localized to the rhoptry bulb tend to be species specific. Since it has been proposed that rhoptry neck proteins, such as RON2, RON4, and RON5, are discharged to form the tight junction between parasites and host cells, this invasion mechanism could be conserved across the Apicomplexa phylum. Recently it was reported that the *Toxoplasma* genome contains paralogous genes for RON2 and AMA1, selectively expressed in sporozoites, suggesting that *Toxoplasma* has developed stage-specific invasion mechanisms (Poukchanski et al., 2013). Since the *Plasmodium* genome contains only one set of genes encoding RON2 and AMA1, it would be interesting to examine whether the rhoptry neck protein complex is formed during sporozoite invasion of mosquito salivary glands and mammalian hepatocytes. In contrast, it has been revealed that many of the *Toxoplasma* rhoptry bulb proteins are involved in interaction with host signaling pathways (Lim et al., 2012) and interference with host immunity after invasion (Fentress et al., 2010; Ong et al., 2010; Steinfeldt et al., 2010; Yamamoto et al., 2011; Niedelman et al., 2012). These mechanisms are likely host cell-specific adaptations, and therefore the expression of rhoptry bulb proteins might be variable among parasites species and stages. As most rhoptry proteins are refractory to gene disruption in both *Plasmodium* and *Toxoplasma*, the sporozoite-specific gene knockdown system by promoter swapping is a powerful tool to reveal functions of rhoptry proteins in sporozoites (Ishino et al., 2019). To differentiate the conserved- or specific- mechanisms of invasion among species or infective stages would give clues to understand the comprehensive molecular basis of parasite infection.

The mRNA amounts of ASP/RON1 and RON3 are clearly greater in sporozoites than in schizonts. This suggests that ASP/RON1 and RON3 might mainly have roles or additional roles in sporozoites. Indeed, unlike other rhoptry proteins, ASP/RON1 is dispensable for parasite proliferation during the intraerythrocytic stage in *P. berghei* (Bushell et al., 2017). Understanding its function in sporozoites would reveal the different contributions of rhoptry proteins depending on infective stages or target host cells.

This is the first profiling data showing the expression and localization of rhoptry proteins in both merozoites and sporozoites by immuno-electron microscopy. By generation of transgenic parasite lines expressing c-Myc tagged rhoptry proteins, comprehensive localization analyses by immuno-electron microscopy could be performed using anti-c-Myc antibodies, thus overcoming the difficulty to obtain specific and high titer antibodies against *Plasmodium* proteins. The c-Myc tag, a peptide of 10 amino acids and ~1,200 Da, is relatively small and unlikely to interfere with protein folding and function following addition to the C-terminus of target proteins that do not possess a C-terminal GPI-anchor domain. All the transgenic parasite lines, in which native rhoptry proteins were replaced by c-Myc tagged rhoptry proteins, proliferate normally during the intra-erythrocytic stage. Considering that most rhoptry proteins are refractory to gene deletion due to their necessity for parasite proliferation in the blood stage (Tufet-Bayona et al., 2009; Giovannini et al., 2011; Counihan et al., 2013; Bushell et al., 2017), this indicates that c-Myc tagging at the C-terminus of each protein doesn't affect rhoptry protein localization and function. This c-Myc tagging application combined with IEM will facilitate the observation of protein trafficking and discharge; for example, by overcoming the difficulties in producing specific antibodies with high titer.

In merozoites of *P. berghei* ANKA, as well as in *P. falciparum*, RON2, RON4, RON5, RALP1, and ASP /RON1 are localized to the rhoptry neck, while RAP1, RON3, RAMA, RhopH1A, RhopH2, and RhopH3 are localized to the rhoptry bulb. It has been proposed that sub-localization of rhoptry proteins reflects the order of discharge during merozoite invasion; that is, rhoptry neck proteins are secreted prior to invasion and followed by release of rhoptry bulb proteins (Zuccala et al., 2012). In this study we demonstrate that rhoptry neck proteins are also expressed in sporozoites; however, their localization, except for ASP/RON1, is not restricted to the rhoptry neck. This observation is in good agreement with reports demonstrating that the localization of the rhoptry proteins RON11 and RON12 differ between merozoites and sporozoites (Bantuchai et al., 2019; Oda-Yokouchi et al., 2019). RON2 in sporozoites is required for salivary gland invasion as well as hepatocyte infection (Ishino et al., 2019), which is not the case for RON2 in merozoites involved in erythrocyte invasion, supporting that localization in sporozoite rhoptries might be different from that in merozoites. Further analyses will reveal whether the protein secretion mechanisms and/or timing is different between merozoites and sporozoites. In addition, the same strategy can be used to address protein secretion mechanisms, such as how proteins might be transported to but differentially localized within rhoptries in sporozoites vs. merozoites.

## Data Availability

All datasets generated for this study are included in the manuscript/Supplementary Files.

## Ethics Statement

All animal experimental protocols were approved by the Institutional Animal Care and Use Committee of Ehime University and the experiments were conducted according to the Ethical Guidelines for Animal Experiments of Ehime University.

## Author Contributions

NT, MTo, and TI conceived and designed the experiments. NT, MN, MTa, MB, KM, MTo, and TI performed the experiments and analyzed the data. MN, MTo, and TI wrote manuscript. NT, MTa, MB, KM, and TT critically reviewed it.

### Conflict of Interest Statement

The authors declare that the research was conducted in the absence of any commercial or financial relationships that could be construed as a potential conflict of interest.

## Abbreviations

AMA1: apical merozoite protein 1
*An*: *Anopheles*
ASP: apical sushi protein
ef1α: elongation factor 1A
GPI: glycosylphosphatidylinositol
IFA: immunofluorescent assay
IEM: immuno-electron microscopy
*Pb*: *P. berghei*
RAMA: rhoptry associated membrane antigen
PBS-MT: PBS containing 5% non-fat dry milk and 0.01% Tween 20
PBS-BT: PBS containing 0.4% Block Ace and 0.01% Tween 20
PVM: parasitophorous vacuole membrane
RALP1: rhoptry-associated leucine zipper-like protein 1
RAP1: rhoptry-associated protein 1
RhopH: high-molecular mass rhoptry protein
RON2: rhoptry neck protein
RT-PCR: reverse transcription-PCR
SPECT2: sporozoite protein essential for cell traversal 2.
