# Supplementary material for: Expression and Localization Profiles of Rhoptry Proteins in Plasmodium berghei Sporozoites
Source: Front Cell Infect Microbiol. 2019 Sep 10;9:316. doi: 10.3389/fcimb.2019.00316 (PMC6746830; doi:10.3389/fcimb.2019.00316)
Supplement: Supplementary file 1 [file Table_1.docx]

Supplementary Material

# Supplementary Figure legends

**Supplementary Figure S1. Scheme for plasmid construction to generate transgenic parasites expressing c-Myc tagged rhoptry proteins.** Genomic DNA fragments with over 1500 bp ending at the C-terminus of each encoded protein were amplified using specific primers (see supplementary table). Nucleotide numbers from the first methionine are indicated. (A) Amplified fragments were inserted into the transgenic vector containing c-Myc coding sequence followed by the 3’UTR of the dhfr/ts gene (pL0033, obtained from BEI) at SacII and NcoI sites. Purified plasmids were linearized with the indicated restriction enzymes prior to transfection to parasites. (B) The NdeI recognition site in pL0033 plasmid was deleted (pL0033-NdeI) by inverse PCR before cloning the amplified DNA fragments corresponding to RON5 and RALP1. The plasmids were linearized at the NdeI site present in the inserted DNA fragments. (C) The amplified DNA fragments corresponding to RON3, RON4, and RON6 were inserted into pGEM T-easy vectors. Inserted DNA fragments were modified by inverse PCR as indicated to introduce XbaI recognition sites (RON3 and RON4) or to delete a NcoI recognition site (RON6). Each DNA fragment was then inserted into the pL0033 vector at the SacII and NcoI sites. Purified plasmids were linearized by XbaI or BamHI, as indicated, prior to transfection into WT-GFP schizonts.

**Supplementary Figure S2. Schematic representation of single crossover homologous recombination to integrate transgenic vectors.** In vitro cultured schizont-enriched parasites of WT-GFP were transfected by electroporation with linearized transgenic vectors containing a human DHFR expression cassette as a drug resistant marker. As a result of homologous recombination the coding region corresponding to the C-terminus of target proteins fused in-frame to the c-Myc tag sequence were integrated in the locus. The DNA integrated parasites were selected by pyrimethamine treatment and cloned. PCR genotyping was performed to confirm correct DNA integration in the target locus.

**Supplementary Figure S3. Schematic representation of the secondary structure of ASP/RON1, RAMA, and RON3.** Since it is predicted that ASP/RON1 and RAMA contain GPI-anchor domains (indicated as green boxes) at their C-terminus, c-Myc tagging to the C-terminus might disturb their correct localization and consequently functions. Recombinant proteins corresponding to the regions shown as bars were synthesized as GST fusion proteins using the wheat germ cell free system. Purified recombinant proteins were used as antigens to immunize rabbits. It was reported that RAMA contains repeated peptides which are processed during merozoite maturation. This was confirmed by Western blot analyses (Figure 2) in *P. berghei* merozoites and sporozoites. SP, signal peptide; GPI, GPI-anchor domain, Coiled-coil, coiled-coil region.

**Supplementary Table S1**

**Primers for RT-PCR**

| Primer Name | Sequence (5’-3’) |
| --- | --- |
| PbASP RT-F | GAAGTAGTACAGCATGACTTCC |
| PbASP RT-R | CAGCTAGCAATGCACCTTCTA |
| PbRON2 RT-F | CGTCTACATCGGCCTTTATTC |
| PbRON2 RT-R | GCGATAGCATGTGTTGTAAATTGG |
| PbRON3 RT-F | TGTGTTTGGAAGAGGATGATGA |
| PbRON3 RT-R | GCTTGACTGCTCCGATTAATG |
| PbRON4 RT-F | GCTACTTATTAGAGAGCGAAAAC |
| PbRON4 RT-R | CATGTGCTAATACGTTGTGTG |
| PbRON5 RT-F | ATGCAAGGAACTGACAAGCA |
| PbRON5 RT-R | ACAACACTGTCTGGCCTCAC |
| PbRON6 RT-F | TATGAAGGTGTCAGATGCTGG |
| PbRON6 RT-R | ACTTCTCTAACCCTCGCATTG |
| SPECT2 RT-F | CCACATTACATCATGTAGACCAG |
| SPECT2 RT-R | TGATCTATTAACTGCACCATGGA |
| PbEF1-alfa RT-F | TGGAACCACCCAAAAGACCA |
| PbEF1-alfa RT-R | ACAACAGCAGATGGAGCGAA |
| PbRALP RT-F | GTTCACAAAGAGTTGATTTAGCTC |
| PbRALP RT-R | GTAACTCCCCTTGTCCTTGA |
| PbRAMA RT-F | TGACGATATGATGGAGTTAGAAAG |
| PbRAMA RT-R | GAGCTAGAGTTAGACTCCTTCG |
| PbRAP1 RT-F | CAAGTGCCGATTTGCCAGATTA |
| PbRAP1 RT-R | TTTGGTAGAATGCTGAAATACGC |
| PbRhopH1A RT-F | CTCACAGGCGTAATGACTCG |
| PbRhopH1A RT-R | GAACGTTGTATGCAGAACGGAG |
| PbRhopH2 RT-F | ATCATTATGGATGCATGCAGC |
| PbRhopH2 RT-R | CATTTCGGCAATGTTCTTCGG |
| PbRhopH3 RT-F | AATATGCAAGGTCATGTGGAGC |
| PbRhopH3 RT-R | ACGGACTAGACGGGTTAGATAAA |

**Primers for c-Myc vector construction**

| Primer Name | Sequence (5’-3’) |
| --- | --- |
| PbRON3::c-Myc F SacII | TCCccgcggAAGAGGAAGATTTAATGCCTCAATCA |
| PbRON3::c-Myc R NcoI | CATGccatggATGGAATGTATAATTGTTGTTTTTTTGTTATTC |
| PbRON4::c-Myc F SacII | TCCccgcggGATAAAGGAATGAATAGCATTACCG |
| PbRON4::c-Myc R NcoI | CATGccatggATAAATCATCAAAAATGGCTTTCTCAAC |
| PbRON5::c-Myc F SacII | TCCccgcggGTTGGAGGTAATAATAGAACAAAATTCA |
| PbRON5::c-Myc R NcoI | CATGccatggAAGGTATTCTTGTATGAACAATAATTTCA |
| PbRON6::c-Myc F SacII | TCCccgcggGAGAAAATGATGTTGCTGAAATAGA |
| PbRON6::c-Myc R NcoI | CATGccatggATTTGTTTGATGGTAACTCAGATTTTTG |
| PbRALP::c-Myc F SacII | TCCccgcggAATATGAAATAATGTAAAGAAGGTGCAT |
| PbRALP::c-Myc R NcoI | CATGccatggAAATTAGATTAGAAAGCATTGCATATAATTTC |
| PbRAP1::c-Myc F SacII | TCCccgcggTTCATTAAAGGATAGGGATTACGC |
| PbRAP1::c-Myc R NcoI | CATGccatggAGAATACACGATCATAAATAAAAAAGTCC |
| PbRhopH1A::c-Myc F SacII | TCCccgcggTTATACACGTAAACTCTTTTATGCAACT |
| PbRhopH1A::c-Myc R NcoI | CATGccatggATATTATGCTCCTTTGGTTCAGG |
| PbRhopH2::c-Myc F SacII | TCCccgcggGATGTTCATAAAGTCGATGCAAAG |
| PbRhopH2::c-Myc R NcoI | CATGccatggATTTGCTCTCTAAAAGCTCTATGTTC |
| PbRhopH3::c-Myc F SacII | TCCccgcggATGTATGTGGGTTTAATGAGAAAGC |
| PbRhopH3::c-Myc R NcoI | CATGccatggATAATAAATTTTTATCTTCATCGGTATAAGG |

**Primers for recombinant protein production**

| Primer Name | Sequence (5’-3’) |
| --- | --- |
| PbASP recF EcoRV | ATCgatatcGGCGTTAACTTTTTAAATAGATTTAATAG |
| PbASP recR BamHI | CCggatccCTAAGGGTCATTTTTAAGTTTATAAATATTTTCG |
| PbRON3 rec F2-XhoI | CCGctcgagGACGATAATTTGTCAAACGCATCA |
| PbRON3 rec R1-BamHI | CCggatccTATCTGAATATGTCTTTTTTAGGAAC |
