# Supplementary material for: Expression and Localization Profiles of Rhoptry Proteins in Plasmodium berghei Sporozoites
Source: Front Cell Infect Microbiol. 2019 Sep 10;9:316. doi: 10.3389/fcimb.2019.00316 (PMC6746830; doi:10.3389/fcimb.2019.00316)
Supplement: Supplementary file 2 [file Presentation_1.PPTX]

## Slide 1
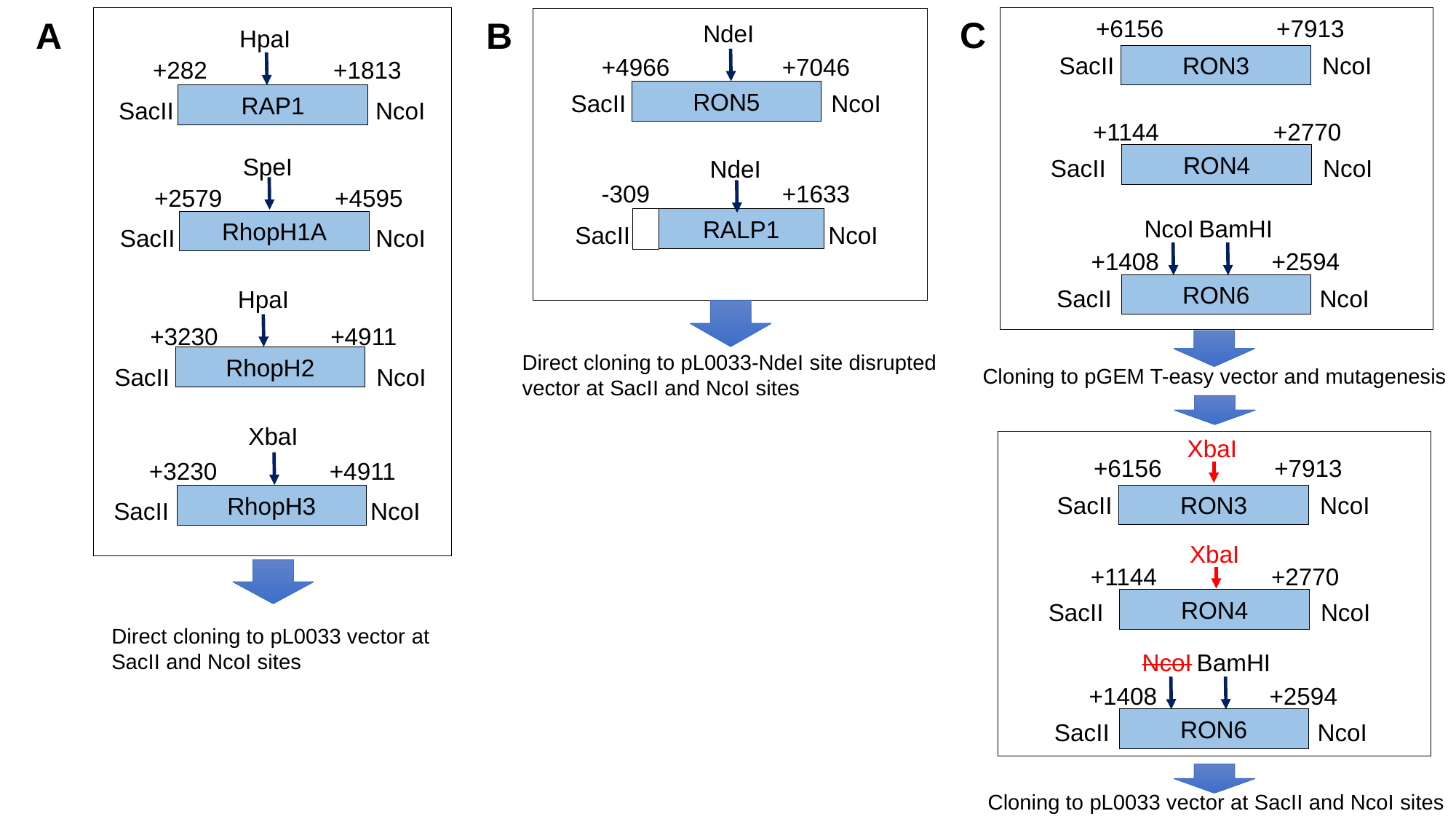

+6156
+7913
RON3
C
B
A
NdeI
+4966
+7046
RON5
HpaI
+282
+1813
RAP1
SacII
NcoI
+1144
+2770
RON4
SacII
NcoI
SacII
NcoI
SacII
NcoI
SpeI
+2579
+4595
RhopH1A
NdeI
-309
+1633
RALP1
NcoI
BamHI
SacII
NcoI
SacII
NcoI
+1408
+2594
RON6
SacII
NcoI
HpaI
+3230
+4911
RhopH2
Direct cloning to pL0033-NdeI site disrupted vector at SacII and NcoI sites
SacII
NcoI
Cloning to pGEM T-easy vector and mutagenesis
XbaI
+3230
+4911
RhopH3
XbaI
+6156
+7913
SacII
NcoI
RON3
SacII
NcoI
+1144
+2770
RON4
XbaI
SacII
NcoI
Direct cloning to pL0033 vector at SacII and NcoI sites
NcoI
BamHI
+1408
+2594
RON6
SacII
NcoI
Cloning to pL0033 vector at SacII and NcoI sites

## Slide 2
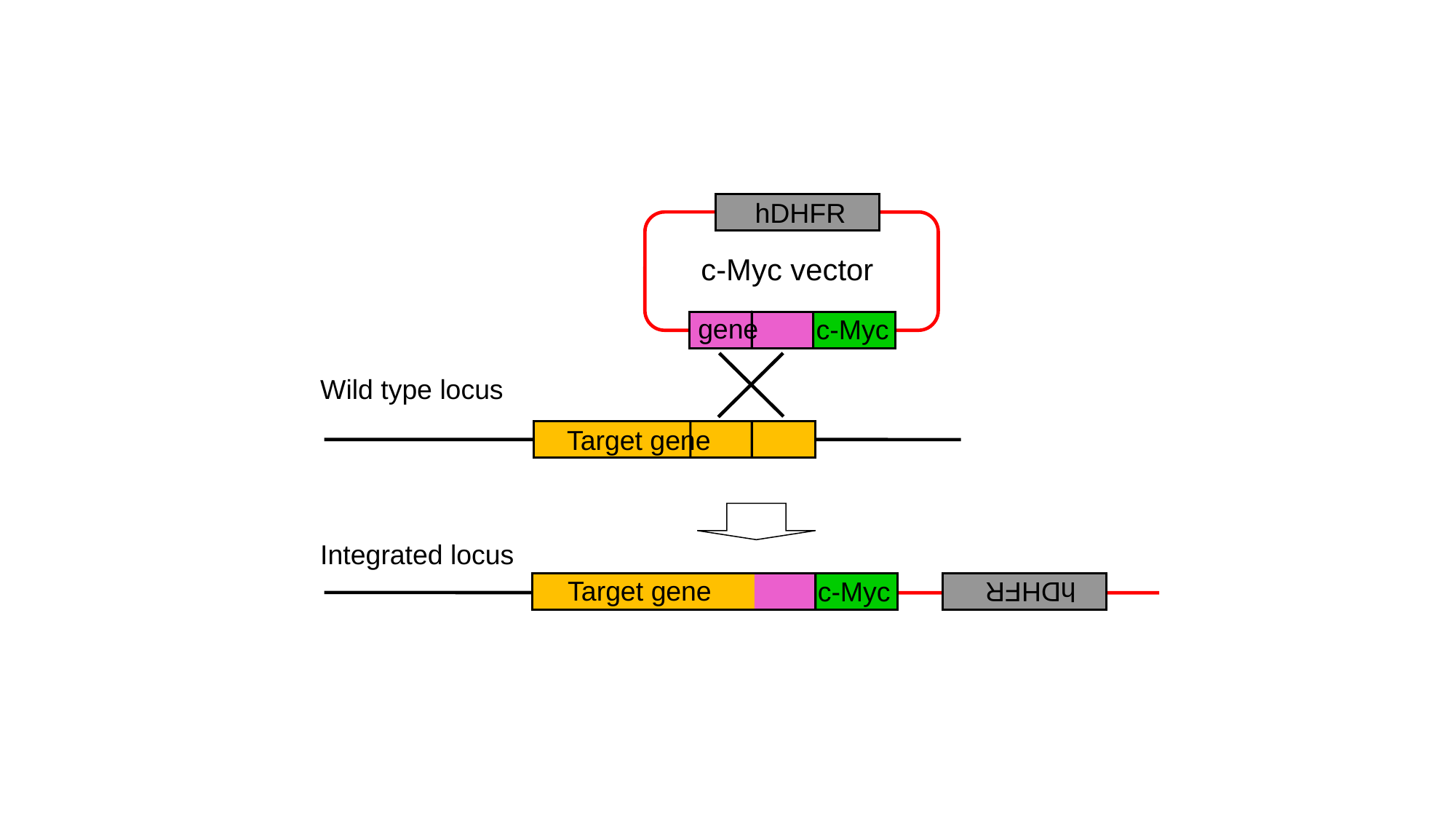

hDHFR
c-Myc vector
gene
c-Myc
Wild type locus
Target gene
Integrated locus
Target gene
c-Myc
 hDHFR

## Slide 3
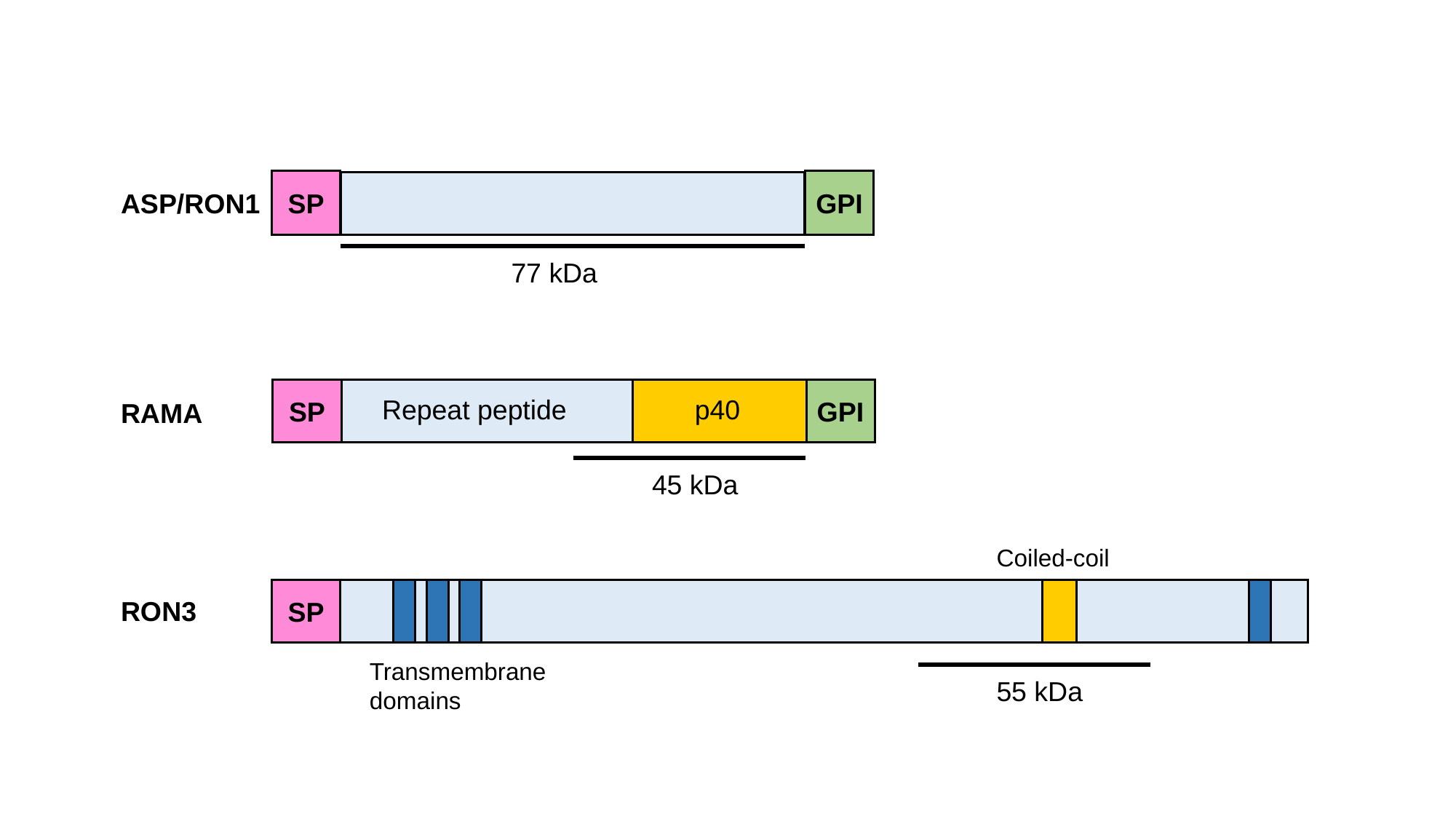

SP
GPI
ASP/RON1
77 kDa
SP
GPI
Repeat peptide
p40
RAMA
45 kDa
Coiled-coil
SP
RON3
Transmembrane
domains
55 kDa
